# Supplementary material for: Jointly Embedding Multiple Single-Cell Omics Measurements
Source: Algorithms Bioinform. Author manuscript; Available in PMC 2021 Oct 7. (PMC8496402; doi:10.4230/LIPIcs.WABI.2019.10)
Supplement: 1 [file NIHMS1060040-supplement-1.pdf]

A Supplement

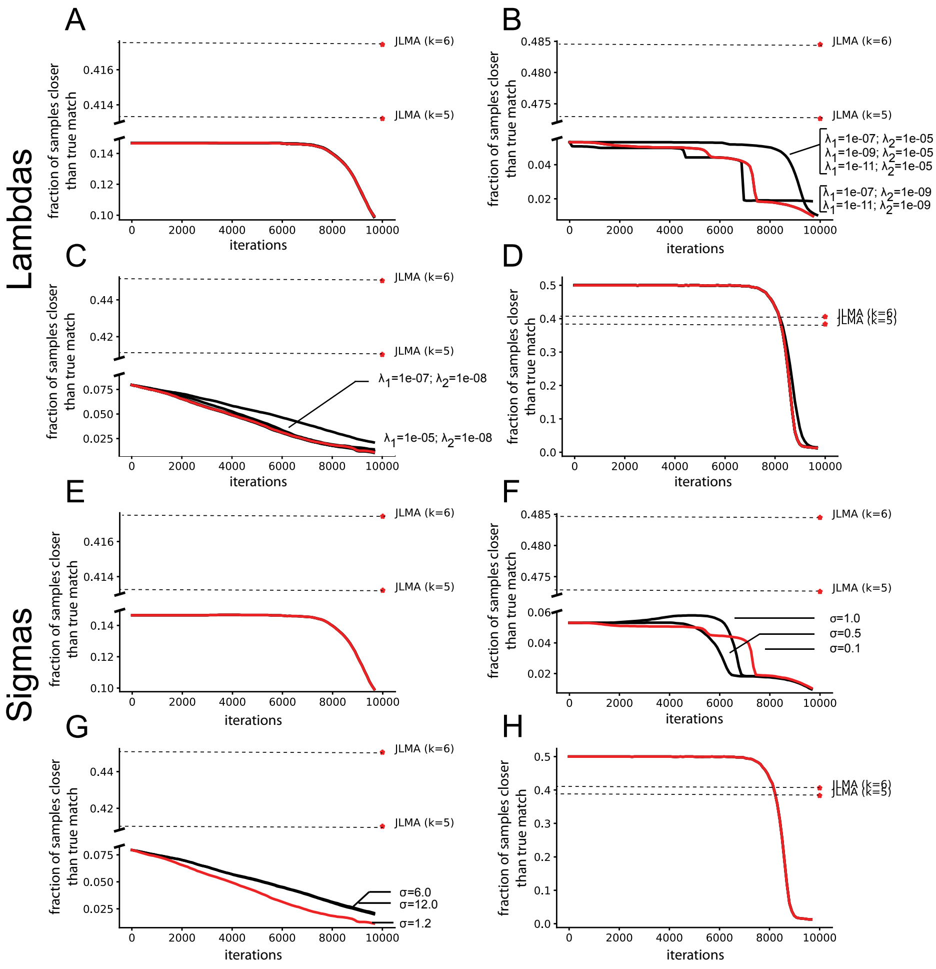

Figure 3 Testing sensitivity to hyperparameters. (A–D) The performance of MMD-MA when the trade-off parameters λ_1 and λ_2 are set differently in the three numerical simulations and the one real-world application, respectively. In each case, eight settings were chosen, and the plot only shows curves (black color) that differ from the one produced by the selected parameters (red color). (E–H) The performance of MMD-MA when σ is set differently in the three numerical simulations and the one real-world application, respectively. Again, only settings that yield results different from the selected results are shown.

Table 3 Hyperparameter values investigated for each data set.

Data set	λ_1	λ_2
Simulation 1	1e-04	1
	1e-04	1e-02
	1e-04	1e-04
	1e-06	1
	1e-06	1e-02
	1e-06	1e-04
	1e-08	1
	1e-08	1e-02
	1e-08	1e-04
Simulation 2	1e-07	1e-05
	1e-07	1e-07
	1e-07	1e-09
	1e-09	1e-05
	1e-09	1e-07
	1e-09	1e-09
	1e-11	1e-05
	1e-11	1e-07
	1e-11	1e-09
Simulation 3	1e-03	1e-04
	1e-03	1e-06
	1e-03	1e-08
	1e-05	1e-04
	1e-05	1e-06
	1e-05	1e-08
	1e-07	1e-04
	1e-07	1e-06
	1e-07	1e-08
Methyl-Expr	1	1e-04
	1	1e-06
	1	1e-08
	1e-02	1e-04
	1e-02	1e-06
	1e-02	1e-08
	1e-04	1e-04
	1e-04	1e-06
	1e-04	1e-08
